# Rapid and sensitive on-site detection of SARS-CoV-2 RNA from environmental surfaces using portable laboratory devices

**DOI:** 10.1128/spectrum.00456-23

**Published:** 2023-10-04

**Authors:** Kouichi Kitamura, Minami Kikuchi Ueno, Hiromu Yoshida

**Affiliations:** 1 Department of Virology II, National Institute of Infectious Diseases, Musashimurayama-shi, Tokyo, Japan; Laboratory Corporation of America Holdings, Burlington, North Carolina, USA

**Keywords:** SARS-CoV-2, poliovirus, enterovirus, noroviruses, on-site detection, environmental surface

## Abstract

Monitoring severe acute respiratory syndrome coronavirus 2 (SARS-CoV-2) RNA on inanimate surfaces plays a crucial role in environmental surveillance, enabling the indirect detection of infected individuals and facilitating rapid infection control responses. However, developing a practical on-site, end-to-end method for detection remains a challenge. In this study, we established protocols for on-site detection of SARS-CoV-2 RNA on surfaces using two portable laboratory devices: The PicoGene PCR1100 and Bento Lab. Test particles comprised of inactivated viral particles containing partial sequences of SARS-CoV-2, type 1 poliovirus, coxsackie virus A16, and norovirus genogroups I and II were applied to reference materials as simulated contaminants. The direct detection method with the PicoGene PCR1100 achieved a recovery efficiency of 35.1% for the dried reference material. Furthermore, the Bento Lab, equipped with a microcentrifuge for on-site RNA purification, successfully detected a low concentration of inactivated SARS-CoV-2 reference standard. The recovery efficiency of the on-site RNA purification method on plastic, stainless steel, glass, and polyvinyl chloride surfaces ranged from 15.5% to 21.2%. Through the combination of these portable devices, SARS-CoV-2 RNA was detected within 1 h of swab sampling. This study contributes to the on-site risk assessment of infectious diseases by enabling the rapid detection of viral genomes.

**IMPORTANCE:**

This study presents the development of a highly sensitive on-site method for detecting severe acute respiratory syndrome coronavirus 2 (SARS-CoV-2) RNA on various surfaces, including doorknobs and tables. Identifying SARS-CoV-2 RNA on these surfaces can be crucial in guiding decision-making for implementing non-pharmaceutical interventions, such as zoning strategies, improving ventilation, maintaining physical distancing, and promoting increased hand hygiene practices. Moreover, the on-site detection system can facilitate the swift initiation of mitigation responses in non-laboratory settings, including long-term care facilities and schools. The protocols established in this study offer a comprehensive approach for achieving both sensitivity and rapidity in on-site SARS-CoV-2 RNA detection. Furthermore, since the RT-qPCR assay serves as the gold standard for detecting viral RNAs, the developed protocol holds potential for application to other viruses, including enteroviruses and noroviruses.

## INTRODUCTION

Severe acute respiratory syndrome coronavirus 2 (SARS-CoV-2) can be transmitted by symptomatic and asymptomatic infected individuals. During the early days of the COVID-19 pandemic, the contaminated surface of fomites was regarded as a significant route of transmission in addition to aerosol transmission (1). The detection of SARS-CoV-2 RNA on various surfaces via quantitative reverse transcription PCR (RT-qPCR) raised concerns about a high risk of transmission. Despite the identification of a significant amount of SARS-CoV-2 RNA on inanimate surfaces, successful viral isolation has been infrequent and mostly unattainable (2 – 6). Although the risk is generally regarded as low, transmission of the virus can still occur through contact with contaminated surfaces (7, 8).

Experimental studies have demonstrated that SARS-CoV-2 virions lose their viability on surfaces within a few days, while the viral RNA can persist for at least 3 weeks (9, 10). Due to the lack of a direct correlation between viral RNA concentration and the presence of intact virions, determining the viability of the virus on surfaces through RT-qPCR has proven challenging. Nonetheless, environmental monitoring plays a valuable role in indirectly detecting the presence of infected individuals within indoor communities, thereby enhancing infection control and response strategies (11, 12). To enable rapid detection in non-laboratory settings such as long-term care facilities and schools, developing an on-site technique for detecting SARS-CoV-2 RNA is necessary.

On-site detection of SARS-CoV-2 at point-of-care testing utilizes various techniques, including rapid immunoassays (13). The loop-mediated isothermal amplification (LAMP) method has been reported to detect viruses in environmental samples (14 – 16). However, RT-qPCR remains the gold standard due to its high sensitivity and specificity (17). The PicoGene PCR1100, a mobile real-time PCR device, has been demonstrated to rapidly detect viral RNAs, including those of SARS-CoV-2 (18 – 21). Unlike conventional real-time PCR machines, PicoGene employs a chip with three distinct temperature regions. The reaction mixture is propelled between these regions through air pressure, enabling rapid real-time PCR detection. However, an end-to-end protocol encompassing sample preparation has yet to be developed for on-site detection. The standard sample preparation method necessitates RNA purification via centrifugation, which is impractical in non-laboratory settings. Although alternative methods, such as simple preparation and direct detection, are available without centrifugation, their sensitivity is comparatively lower as they involve sample dilution.

In this study, we developed a comprehensive on-site detection method using the PicoGene PCR1100 device and a direct detection approach for identifying SARS-CoV-2 on inanimate surfaces. Additionally, we utilized the Bento Lab, which includes a microcentrifuge capable of on-site RNA purification from low-copy viral particles. The study employed artificial particles containing partial sequences of SARS-CoV-2 and enteric viruses, including poliovirus, non-polio-enterovirus, and noroviruses. These enteric viruses are commonly transmitted through fecal-oral infection and contaminated surfaces, making them crucial targets for infection control in healthcare facilities, schools, and in ensuring food safety throughout the supply chain. Furthermore, these viruses serve as reference points to expand the utility of rapid on-site detection devices to other virus species.

## RESULTS

### Sensitivity of SARS-CoV-2 RT-qPCR assay with PicoGene PCR1100

**Fig 1:**
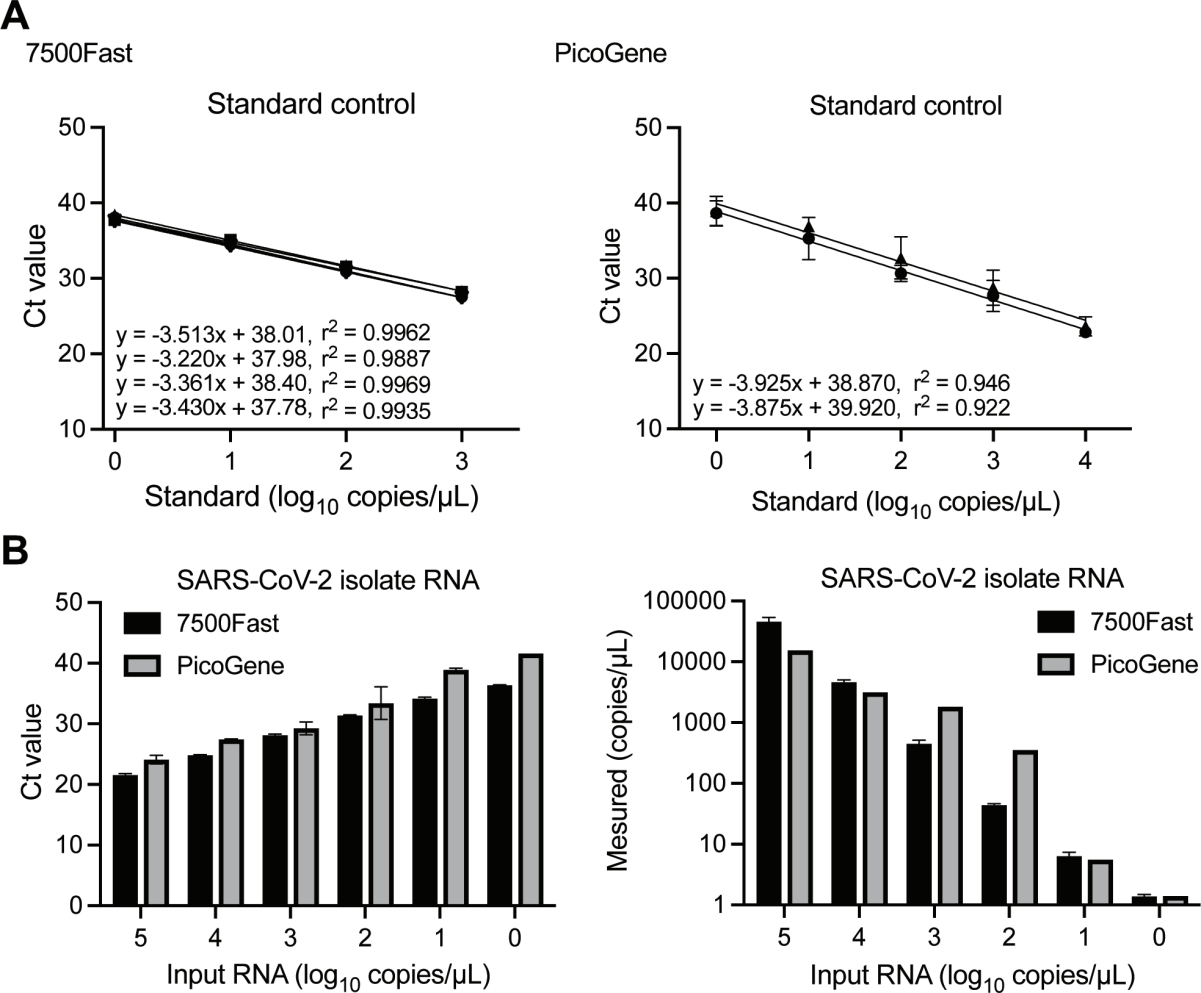
Comparison of SARS-CoV-2 RT-qPCR assays with the ABI 7500 Fast and PicoGene PCR1100. (A) Standard curves of CDC N1/N2 assay. Ct values are presented for 10-fold serial dilutions of RNA standard (1 × 10^4^–1 × 10^0^Copies/µL). The test was carried out four times for ABI 7500 Fast and twice for PicoGene. Linear regression and correlation coefficients are presented. (B) Quantification of SARS-CoV-2-isolate RNA. Ct values and measured copy numbers of the virus RNA are presented. Results are shown as the average of duplicates with standard deviation.

Serially diluted RNA standards were quantified by RT-qPCR Centers for Disease Control and Prevention (CDC) N1/N2 duplex assay (Fig. 1A) to evaluate the PicoGene PCR1100 detection of SARS-CoV-2 RNA in low concentrations. The same assay was performed using a conventional real-time PCR (ABI 7500 Fast) for comparison. The amplification efficiency and correlation coefficient (*r^2^*) of the RT-qPCR standard curve were in an acceptable range of 92.6%–104% and 0.9887–0.9969, respectively (Fig. 1A, left). PicoGene RT-qPCR showed a lower amplification efficiency of 79.8%–81.1% with *r^2^* 0.946–0.922 and the lowest detected concentration (1 copy/µL) compared to those of ABI 7500 Fast (Fig. 1A, right). Subsequently, we evaluated the detection sensitivity of the CDC N1/N2 assay using SARS-CoV-2 RNA isolated from the virus (Fig. 1B). PicoGene PCR1100 detected the lowest concentration of SARS-CoV-2 RNA (1 copy/µL), which was consistent with the results of the RNA standards; however, the cycle threshold (Ct) value was over 40.

### Evaluation of the direct detection method for surface swabs using custom viral particles

**Fig 2:**
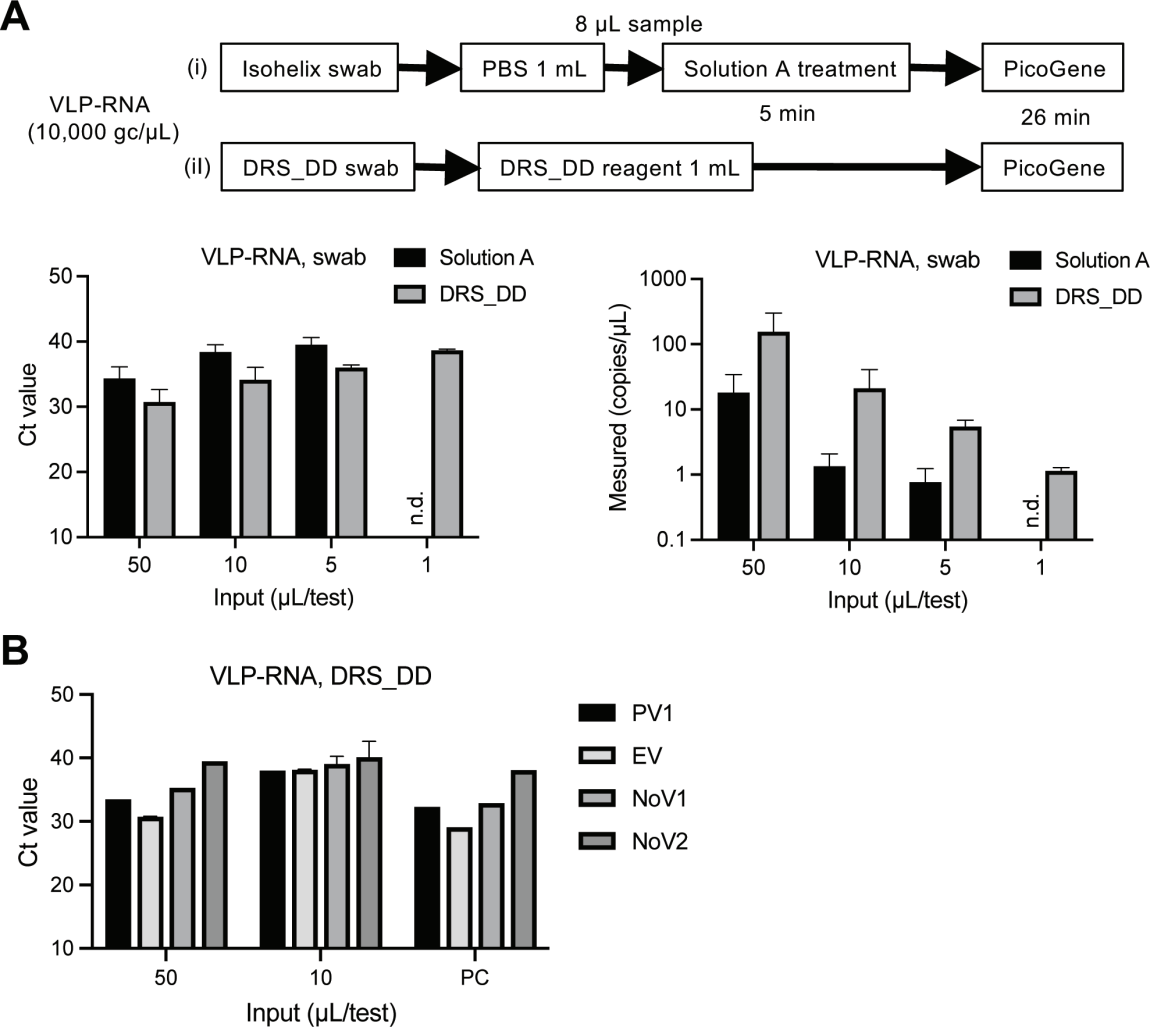
Comparison of sampling methods with VLP-RNA reference material. (A) Various amounts of VLP-RNA were recovered and treated with the (i) simple preparation method with Solution A or (ii) the direct detection method with DRS_DD. Ct values and copy numbers were measured by CDC N1/N2 RT-qPCR assay with PicoGene PCR1100. (B) Detection of enteric virus sequences of poliovirus type 1 (PV1), enterovirus (EV), and norovirus genogroup I and II (NoV1 and NoV2) in VLP-RNA. The samples were prepared with the direct detection method and analyzed with PicoGene. VLP-RNA (1,000 copies/µL) was measured as a positive control. Results are shown as the average of duplicates with standard deviation.

Virus-like particle (VLP)-RNA extraction control (10,000 copies/µL) was used as a process control to determine an effective on-site detection method. The employed reference material comprised VLPs packaged with RNA molecules of custom sequences. In this study, the partial genome sequences of SARS-CoV-2, poliovirus (Sabin 1), enterovirus (coxsackie virus A16), and norovirus genogroups I and II were selected as targets for RT-qPCR tests (22 – 26). After inoculation of the VLP-RNA samples onto the plastic (polystyrene, PS) surface, two sample collection methods were compared. (i) Simple preparation method: samples were recovered with an Isohelix Swab and immersed in phosphate-buffered saline (PBS). The PBS containing VLP-RNA was treated with preparation buffer Solution A (a component of the SARS-CoV-2 Direct Detection RT-qPCR Kit) and then analyzed by RT-qPCR. (ii) Direct detection method: samples were recovered with a DNA/RNA Shield DirectDetect (DRS_DD) swab, immersed in the DRS_DD reagent, and directly used for RT-qPCR (Fig. 2A). The results showed high sensitivity of SARS-CoV-2 detection with PicoGene PCR1100 by the direct detection method using the DRS_DD kit (Fig. 2A). Other target virus sequences in VLP-RNA were also detected with PicoGene PCR1100 and DRS_DD; however, the lower input samples (5 or 1 µL/test) were not detected in the enteric virus RT-qPCR, indicating that sensitivities were lower than those of SARS-CoV-2 detection (Fig. 2B and data not shown).

**Fig 3:**
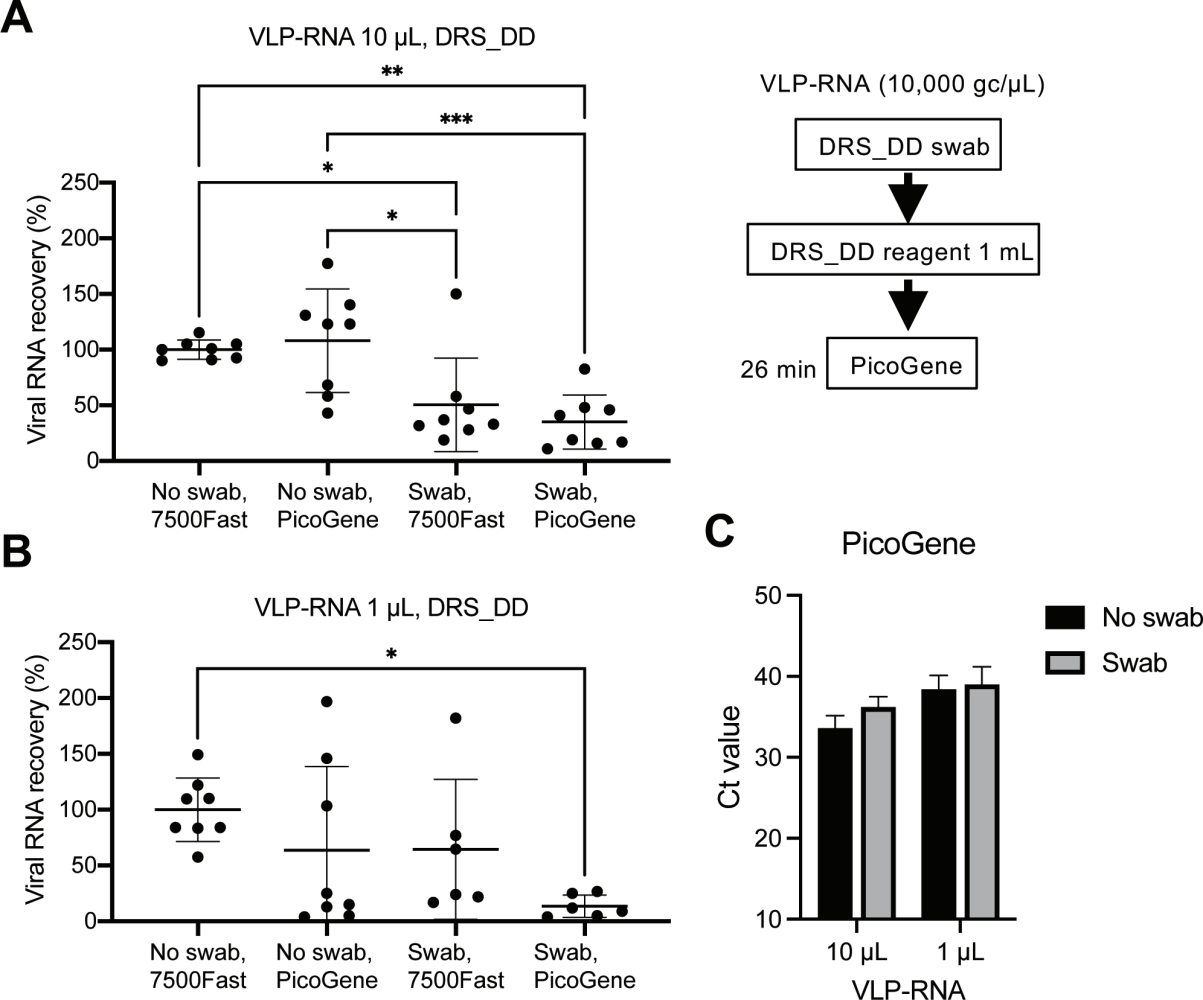
Recovery efficiency of the direct detection method. The indicated amount of VLP-RNA (A: 10 µL, B: 1 µL) was directly added to the DRS_DD reagent (No swab) or inoculated onto a plastic surface and treated with DRS_DD kit (Swab). Copy numbers of VLP-RNA were quantified by CDC N1/N2 RT-qPCR assay with ABI 7500 Fast or PicoGene PCR1100. Each test condition was replicated eight times, and each RT-qPCR assay was tested in duplicate. The average copy number of no swabs with ABI 7500 Fast was 100%. Recovery efficiencies are shown as the average of with standard deviation. Significant differences between conditions were observed in the two-way analysis of variance (ANOVA) test (*P < 0.05, **P < 0.01, ***P < 0.005). (**C)** Ct values measured with PicoGene for the results of A and B.

The evaluation of recovery efficiency for the direct detection method was conducted. To simulate the collection of dried saliva samples, the DRS_DD swab was pre-wet with the reagent and then recovered from the plastic surface where VLP-RNA was spotted. The RNA copy numbers of VLP-RNA were directly added to the DRS_DD reagent (No Swab) and quantified by the ABI 7500 Fast, considered 100% (Fig. 3A and B). In the case of VLP-RNA testing using 10 µL per test, the swab sampling with DRS_DD achieved a viral recovery efficiency of 50.4%, while the overall efficiency of the on-site direct detection method with PicoGene was 35.1% (Fig. 3A). However, for VLP-RNA testing using 1 µL per test, two out of the eight samples were not detected by both the 7500Fast and PicoGene (Fig. 3B). The “no swab” sample, where 1 µL input of VLP-RNA was diluted to 10 copies/µL in the reagent, demonstrates the lowest detection limit achievable by the direct detection method (Fig. 3C).

### On-site detection of inactivated SARS-CoV-2 from surfaces at low concentrations

**Fig 4:**
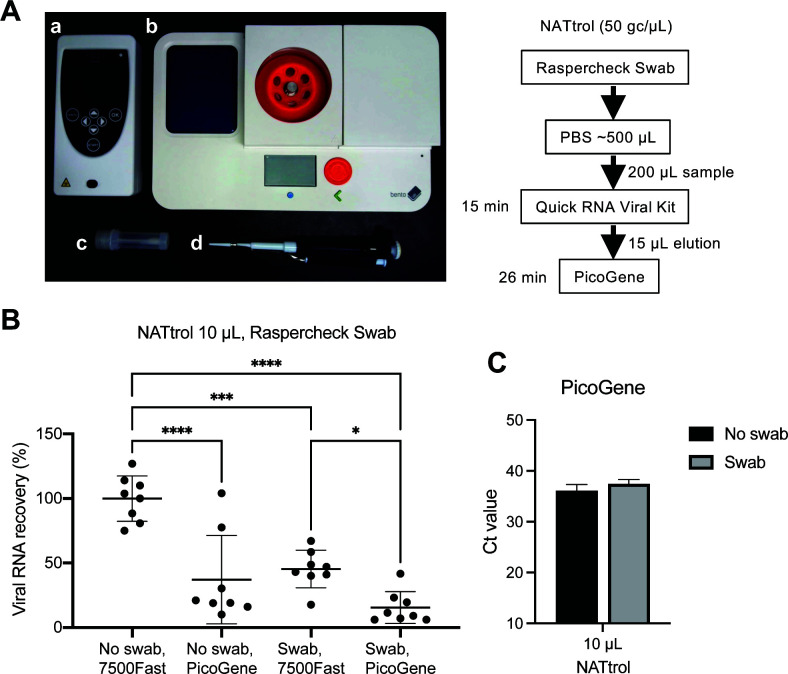
Recovery efficiency of the on-site rapid and sensitive method. Combined with the PicoGene PCR1100, the Bento Lab device was utilized for on-site detection of the low-copy viral RNA. (A) Photograph of portable devices used in this method. (a) PicoGene PCR1100. (b) Bento Lab. (c) Raspercheck Swab. (d) A typical micropipette for size comparison. (B) Ten microliters of NATtrol reference material was directly immersed in PBS (No swab) or inoculated onto a plastic surface and recovered with Raspercheck Swab (Swab). After RNA purification, copy numbers of SARS-CoV-2 were quantified with ABI 7500 Fast or PicoGene PCR1100. Each test condition was replicated eight times, and each RT-qPCR assay was tested in duplicate. The average copy number of no swabs with ABI 7500 Fast was 100%. Recovery efficiencies are shown as the average with standard deviation. Significant differences were observed in the two-way ANOVA test (*P < 0.05, ***P < 0.005, ****P < 0.001). (C) Ct values measured with PicoGene.

**Fig 5:**
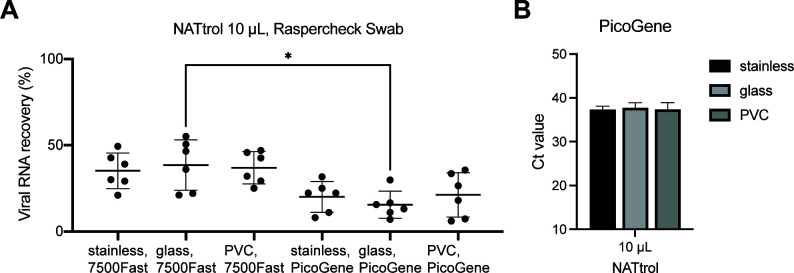
Recovery efficiency of the on-site method from different surface materials. (A) Herein, 10 µL of NATtrol reference material was inoculated onto the surfaces of indicated materials (stainless steel, glass, and polyvinyl chloride). After the Raspercheck Swab sampling and RNA purification with Bento Lab, copy numbers of SARS-CoV-2 were quantified with ABI 7500 Fast and PicoGene PCR1100. Each test condition was replicated six times, and each RT-qPCR assay was tested in duplicate. The average copy number of no swabs with ABI 7500 Fast in Fig. 4B was 100%. Recovery efficiencies are shown as the average of quadruplicate with standard deviation. A significant difference was observed between two real-time PCR instruments for one material in the two-way ANOVA test (*P < 0.05). (B) Ct values measured with PicoGene.

Nucleic acid testing control (NATtrol), an inactivated SARS-CoV-2 reference standard, was used as the process control at low concentrations (50 copies/µL). After recovering the NATtrol sample from the plastic surface with Raspercheck Swab, viral RNA was concentrated by an additional RNA purification step using the Quick RNA Viral Kit and Bento Lab microcentrifuge (Fig. 4A). The recovery efficiency for this on-site sampling/concentration method was 45.4%. The total efficiency of on-site detection with PicoGene was 15.5% (Fig. 4B and C). We also tested this method with various surface materials. The total recovery efficiencies from stainless steel, glass, and polyvinyl chloride were 20.0%, 15.5%, and 21.2%, respectively (Fig. 5).

## DISCUSSION

In this study, we developed on-site sample preparation methods and a mobile real-time PCR assay to detect viral RNA on environmental surfaces. We observed that the quantification of PicoGene RT-qPCR was relatively less reliable compared to the conventional real-time PCR instrument, as indicated by the evaluation of standard curves ranging from 1 to 10,000 copies/µL (Fig. 1A). This lower linearity could be attributed to the “ultra-rapid” thermal cycling utilized by PicoGene (18 – 20). Even the “fast mode” of the conventional real-time PCR instrument showed a tendency toward lower linearity under fast PCR conditions (27, 28). However, despite the lower linearity, the limit of detection achieved by PicoGene with the CDC N1/N2 duplex assay was 1 copy/µL (Fig. 1A and B), which is comparable to that of the ABI 7500 Fast (29). This suggests that the sensitivity of PicoGene for detecting SARS-CoV-2 has been improved compared to previous studies (20, 21). In a previous study (21), a multi-channel assay employing FAM and Cy5 channels was used to detect SARS-CoV-2 along with RNase P as an internal control in clinical samples. However, our preliminary tests found that the multi-channel assay resulted in lower detection sensitivity. Hence, we opted for a single-channel approach in this study. Considering that the sensitivity of the detection method is more crucial than precise quantification when monitoring viral RNA on environmental surfaces, PicoGene RT-qPCR is useful for rapid screening to detect SARS-CoV-2 RNA on inanimate surfaces. Furthermore, the reaction time for PicoGene is less than 30 min which is shorter than required for conventional RT-qPCR. This rapid turnaround time further enhances the practicality and efficiency of using PicoGene for on-site detection.

The 5 µL of VLP-RNA was not detected with enteric virus RT-qPCR, indicating that the detection sensitivities for these viruses were lower than that of SARS-CoV-2 (Fig. 2B). Further studies are required to optimize RT-qPCR conditions in PicoGene for these viruses.

The total efficiency of the on-site direct detection method for SARS-CoV-2 using PicoGene was 35.1% when 10 µL of VLP was spotted and dried (Fig. 3A). Considering the swab recovery efficiency of 50.4%, the concentration of target molecules in the reagent would be less than 7 copies/µL for VLP-RNA 1 µL/test, resulting in approximately half of the samples remaining undetected by both the 7500 Fast and PicoGene (Fig. 3B). By employing the on-site RNA purification method with the Bento Lab microcentrifuge, sensitive detection of 500 copies/test of NATtrol could be achieved within 1 h of swab sampling (Fig. 4). Other small centrifuges capable of centrifugation at least 8,000 × *g* could also be utilized. Notably, the Bento Lab also contains a conventional PCR thermal cycler and an agarose gel electrophoresis system, enabling genomic analysis with another portable device, the MinION Nanopore sequencer (30). This opens up the possibility of extending the application to on-site molecular typing of viruses using portable sequencers. The averages of Ct values in the controls (No swab, 7500 Fast) for 10 µL of VLP-RNA (Fig. 3) and NATtrol (Fig. 4) were 30.3 and 32.6, respectively. The calculative RNA concentrations in the reagent were 10 copies/µL for VLP-RNA and 1 copy/µL for NATtrol, roughly equivalent to the difference in the Ct values. Thus, our VLP-RNA can detect SARS-CoV-2 RNA with results comparable to the NATtrol reference standard. The VLP-RNA is a recently available product; further evaluation is required from other research groups. NATtrol SARS-CoV-2 control was previously used in environmental surface studies since it does not contain a high glycerol concentration, making it difficult to dry (15).

Isohelix Swab was originally designed for sampling buccal swabs, but it is also used for collecting environmental microbes in the global metagenomic project and SARS-CoV-2 research (15, 31). Previous studies indicated that the Isohelix swab (rayon) is easier to handle and has higher sensitivity for sample collection than the Copan swab (flocked nylon) (15). DRS_DD is a set of swabs and reagents designed for direct detection after nasopharyngeal sampling. The DRS_DD reagent differs from the DNA/RNA Shield (DRS) reagent widely used as a nucleic acid storage/transport medium (32). In our preliminary experiments, the Isohelix and the DRS reagent combination could not successfully detect the VLP-RNA with PicoGene owing to machine error. According to the manufacturer’s datasheet, the DRS_DD reagent reduced sample viscosity. The viscosity may cause the moving failure of the reaction mixture in the PicoGene chip, suggesting that the use of the DRS_DD reagent is better than DRS for direct detection with the PicoGene device. Raspercheck Swab is a cotton swab pre-wetted with PBS and is larger than the other two swabs. Cotton swabs are widely used to detect norovirus on the surface and are included in the standard protocol ISO 15216 (25). Additionally, the recovery of norovirus with other various swab types was evaluated (33). This large pre-wetted swab is not fit for our direct detection method but can be used for the RNA extraction method with the squeezed sample. Although surface materials may influence the recovery efficiency with Isohelix Swab for SARS-CoV-2 RNA (15), no significant difference was observed in the recovery efficiency with Raspercheck Swab depending on the surface material (Fig. 4 and 5). Further evaluation is required to determine the recovery efficiency of each swab type from various materials.

Plastic and steel are typical surfaces of high-contact objects such as tables, keyboards, toys, door handles, faucets, and sinks. Environmental debris on these surfaces may also influence the recovery and detection efficiency of SARS-CoV-2 under field conditions (15). To address this challenge, the RNA purification step can be incorporated into our protocol, which enhances detection efficiency by reducing debris that could inhibit PCR. Another important consideration is the surface property of the material, whether rough or smooth. Detection from rough surfaces may have lower sensitivity compared to smooth surfaces. However, using a large cotton swab allows for some flexibility, enabling it to adapt and fit into grooved surfaces to a certain extent. In order to validate the practicality of our on-site protocols, it is essential to conduct controlled field tests using actual samples and standard laboratory protocols. Detecting and quantifying pathogens on inanimate surfaces are crucial in assessing the risks associated with infectious diseases. The sensitive on-site protocols developed in this study demonstrate the potential for detecting viral RNA even at low concentrations, thus enabling rapid risk assessment in non-laboratory conditions.

## MATERIALS AND METHODS

### Viral reference materials

To obtain the standard curve of the CDC N1/N2 RT-qPCR assay, the Positive Control RNA (US N1/N2) of the SARS-CoV-2 Direct Detection RT-qPCR Kit (Takara Bio, Kusatsu, Japan) of approximately 10^7^ copies/µL was used. Purified SARS-CoV-2 RNA from the isolate (JPN/TY/WK-521) was obtained via the previously described method (29, 34). RNA obtained from the RT-qPCR standard and isolates were serially diluted with the EASY Dilution (for Real Time PCR) solution (Takara Bio). The VLP-RNA Extraction Control (10,000 copies/µL) (Meridian Bioscience, Cincinnati, OH, USA) contained a custom sequence composed of partial sequences of SARS-CoV-2 (26), type 1 poliovirus (Sabin 1) (24), enterovirus (coxsackie virus A16) (22), and norovirus genogroups I and II (23) listed in Table 1. The NATtrol inactivated SARS-CoV-2 control (50 copies/µL) was purchased from ZeptoMetrix (Buffalo, NY, USA).

**TABLE 1:** Sequences for primers, probes, and custom sequences in VLP-RNA

Target virus	Function	Name	Sequence (5'−3')	Reference
SARS-CoV-2	Forward primer	2019-nCoV_N1-F	GACCCCAAAATCAGCGAAAT	(26)
	Reverse primer	2019-nCoV_N1-R	TCTGGTTACTGCCAGTTGAATCTG	
	Taqman probe	2019-nCoV_N1-P	FAM-ACCCCGCATTACGTTTGGTGGACC-BHQ1	
	VLP-RNA	2019-nCoV_N1	UCUGGUUACUGCCAGUUGAAUCUGA GGGUCCACCAAACGUAAUGCGGGGU GCAUUUCGCUGAUUUUGGGGUC	
	Forward primer	2019-nCoV_N2-F	TTACAAACATTGGCCGCAAA	
	Reverse primer	2019-nCoV_N2-R	GCGCGACATTCCGAAGAA	
	Taqman probe	2019-nCoV_N2-P	FAM-ACAATTTGCCCCCAGCGCTTCAG-BHQ1	
	VLP-RNA	2019-nCoV_N2	GCGCGACAUUCCGAAGAAC GCUGAAGCGCUGGGGGCAAAU UGUGCAAUUUGCGGCCAAUGUUUGUAA	
Poliovirus	Forward primer	Polio1F	CTTCGGTATTTTGGCTGTTAGAGTAGT	(24)
	Reverse primer	Polio1R	GACGCGGGCACCAGACT	
	Taqman probe	Polio1P	FAM-GATCACAACCCGACCAAGGTCACCTCC-BHQ	
	VLP-RNA	Sabin1	GACGCGGGCACCAGACUCUGAUGUG UUUGGGUUUUAGAUACACUCUGAUU UUGGAGGUGACCUUGGUCGGGUUGU GAUCAUUGACUACUCUAACAGC CAAAAUACCGAAG	
Enterovirus	Forward primer	HEV-F	TCCTCCGGCCCCTGA	(22)
	Reverse primer	HEV-R1	AATTGTCACCATAAGCAGCCA	
	Taqman probe	HEV-P1	Cy5-CGG AAC CGA CTA CTT TGG GTG TCC GT-BHQ3	
	VLP-RNA	coxsackie virus A16	AAUUGUCACCAUAAGCAG CCAGUAUAAGAAUAAAAGG AAACACGGACACCCAAAGUA GUCGGUUCCGCUGCAGAGUU GCCCGUUACGACACACUGCCC CCUGGGUCGAGGGUAUGUGCU CCGCAGUUAGGAUUAGCCGCAU UCAGGGGCCGGAGGA	
Norovirus	Forward primer	COG1F	CGYTGGATGCGNTTYCATGA	(23)
	Reverse primer	COG1R	CTTAGACGCCATCATCATTYAC	
	Taqman probe	RING1‐TP(a)-N2	FAM-ATYGCGATCYCCTGTCC-MGB-NFQ	
	VLP-RNA	Norovirus-G1	CCAACCCAACCAUUAUACA UUUGUGAUAGAUGGAGCAA GAAAGGAUUAAGAUGGGGAC CCAAACUCAAAUCAAACAAAAC AUCACCGGGGGUAUUAUUUGGG GAAAUAGUAAAUUCACCUUGGGGG GCUUGCACAAAAUUAUUAAUUAUCC AGGGAUCAAUAGGAUUAACUUGUCC AGCAGUCGCGACUGCUGUCGA AGAACCUGCUACAGGAUCCAUU GCAAGAGGGUCAGAAGCAUUAA CCUCCGGUACCAACUGACCAGCG CCACUAGCGCCAUCCACGCUUGA UGUAGCGUCCUUAGACGCCAUCA UCAUUUACGAAUUCGGGCAGAAG AUCGCGAUCUCCUGUCCACAAUC CGAGGUCAUGGAAGCGCAUCCAGCG	
	Forward primer	COG2F	CARGARBCNATGTTYAGRTGGATGAG	
	Reverse primer	COG2R	TCGACGCCATCTTCATTCACA	
	Taqman probe	RING2AL-TP-N4	FAM-TGGGAGGGSGATCGCRATCT-MGB-NFQ	
	VLP-RNA	Norovirus-G2	UCGACGCCAUCUUCAUUCA CAAAACUGGGAGCCAGAUUG CGAUCGCCCUCCCACGUGCU CAGAUCUGAGAAUCUCAUCC AUCUGAACAUUGGCUCUUG	

### Sample inoculation and collection

**Fig 6:**
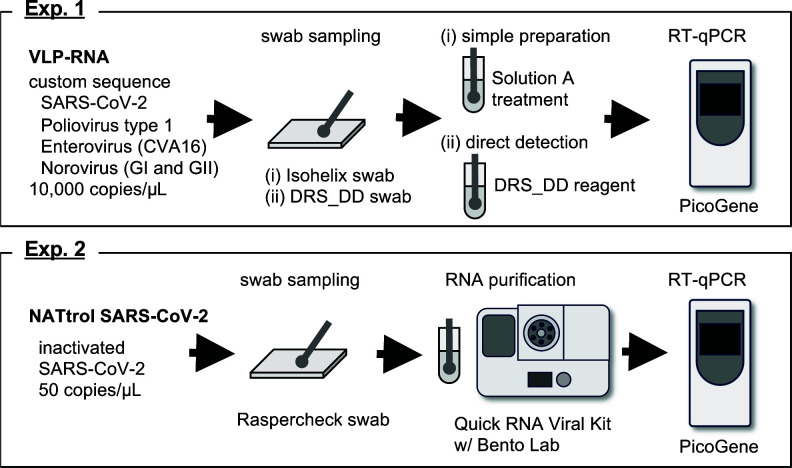
On-site detection methods for detecting viral RNA from environmental surfaces. Experimental method 1 (Exp. 1). VLP-RNA Extraction Control (10,000 copies/µL) contained partial sequences of indicated viruses. Various amounts of VLP-RNA (50, 10, 5, and 1 µL) were inoculated onto the plastic surface and recovered with two sample collection procedures: (i) simple preparation and (ii) direct detection. For (i), samples were collected with Isohelix Swab, resuspended in PBS, and treated with Solution A, an extraction reagent. For (ii), samples were collected using a DNA/RNA Shield DirectDetect (DRS_DD) swab, resuspended in DRS_DD reagent, and directly applied for the RT-qPCR assay with PicoGene (Fig. 2 and 3). Experimental method 2 (Exp. 2). The NATtrol inactivated SARS-CoV-2 control (50 copies/µL) was inoculated onto the plastic (Fig. 4) and other surface materials (stainless steel, glass, and PVC, Fig. 5). Ten microliters of the NATtrol control was recovered with Raspercheck Swab, and the viral RNA was purified from the sample-containing PBS using a Quick RNA Viral Kit and Bento Lab. Purified RNA was used for the RT-qPCR assay with PicoGene.

The methodology in this study is summarized in Fig. 6. Various amounts of VLP-RNA [50, 10, 5, and 1 µL (10,000 copies/µL)] were inoculated onto the plastic surface of Petri dishes. After inoculation, two sample collection procedures were compared: (i) simple preparation and (ii) direct detection. For the simple preparation method, samples were collected with Isohelix Swab (Cell Projects, Kent, UK); 8 µL of VLP-RNA-containing PBS was incubated with 2 µL of Solution A (an extraction reagent of the SARS-CoV-2 Direct Detection RT-qPCR Kit) at 95°C for 5 min, to which 10 µL of nuclease-free water was added. For the direct detection method, samples were collected using a DNA/RNA Shield DirectDetect (DRS_DD) swab, resuspended in DRS_DD reagent (Zymo Research, Irvine, CA, USA), and directly applied for the RT-qPCR assay. Both the Isohelix and DRS_DD swabs were pre-wet with collection reagents. In addition to these two methods, an on-site low-copy SARS-CoV-2 RNA detection method was evaluated. In this method, a Raspercheck Swab (Becton Dickinson, Franklin Lakes, NJ, USA) was used, and 10 µL droplets of the NATtrol control (50 copies/µL) was dried in an uncovered plastic Petri dish for 30 min. The same procedure was performed for the surfaces of stainless steel, glass, and polyvinyl chloride. The dried samples were collected with a pre-wet Raspercheck Swab. The swab was then squeezed into a collection tube to recover the sample-containing PBS. After the collection, viral RNA was purified using a Quick RNA Viral Kit (Zymo Research). The process was performed according to the manufacturer’s instructions; an exception was made for the centrifugation steps, which were performed at 8,000 × *g*, the maximum speed of Bento Lab (Bento Bioworks, London, UK). For the “no swab” control, reference materials were directly added to PBS, and RNA was purified with the Quick RNA Viral Kit.

### RT-qPCR

For RT-qPCR with PicoGene PCR1100 (Nippon Sheet Glass, Osaka, Japan), a 16-µL reaction mixture containing 8 µL of 2 × One Step PrimeScript III RT-qPCR Mix (Takara Bio), 3.2 µL of primer/probe mixture, and 4.8 µL of the sample was used. The sequences and final concentrations of the primer/probe for each target are listed in Table 1. Reaction conditions were programmed as follows: RT at 50°C for 300 s, initial denaturation at 95°C for 15 s, 45 cycles of denaturation at 95°C for 3 s, and annealing/elongation at 60°C for 16 s. For norovirus genogroup I, the annealing and elongation steps were programmed at 56°C for 16 s. The “Sensitivity” parameter was set as “5” for all reactions. The reaction conditions for CDC N1/N2 RT-qPCR with ABI 7500 Fast have been previously reported (29). Reactions with ABI 7500 Fast and PicoGene PCR1100 were duplicated as technical replicates. Viral RNA was not detected in the negative control group.

### Statistical analyses

Two-way analysis of variance followed by multiple comparisons using GraphPad Prism 9 (GraphPad Software, San Diego, CA, USA) was performed to compare the detection efficiency of each method. Statistical significance was set at *P* < 0.05.
